# Hepatoprotective effects of gentisic acid through its anti-oxidant properties in nicotinamide-streptozotocin induced diabetic mice

**DOI:** 10.22038/IJBMS.2023.70659.15359

**Published:** 2023

**Authors:** Reza Noei Razliqi, Elnaz Harooni, Niloofar Neisi, Maryam Jafar Sameri, Akram Ahangarpour

**Affiliations:** 1 Student Research Committee, Ahvaz Jundishapur University of Medical Sciences, Ahvaz, Iran; 2 Infectious and Tropical Diseases Research Center, Health Research Institute, Department of Medical virology, the School of Medicine, Ahvaz Jundishapur University of Medical Sciences, Ahvaz, Iran; 3 Department of Physiology, Abadan University of Medical Sciences, Abadan, Iran; 4 Department of Physiology, Persian Gulf Physiology Research Center, Medical Basic Sciences Research Institute, Ahvaz Jundishapur University of Medical Sciences, Ahvaz, Iran

**Keywords:** Gentisic acid, Liver failure, Nuclear factor E2-related- factor 2, Oxidative stress, Type 2 diabetes mellitus

## Abstract

**Objective(s):**

Type 2 diabetes mellitus (T2DM) is a common metabolic disorder that causes many complications. Liver failure is one of the complications of T2DM. Oxidative stress plays a major role in the development and progression of T2DM-induced liver injury. Gentisic acid (GA) is a metabolite of aspirin and also a phenolic compound found in natural sources that is a highly effective antioxidant and free radical scavenger. So, in this study, the potential preventive benefits of GA against liver damage induced by T2DM were explored.

**Materials and Methods:**

This study was conducted on 24 adult male mice. T2DM was induced by intraperitoneal injection of a single dose of streptozotocin (at a dose of 65 mg/kg), 15 min after the injection of nicotinamide (at a dose of 120 mg/kg). The grouping was as follows: 1) Normal Control Group; 2) Diabetic Control Group; 3) Positive Control Group: received metformin (150 mg/kg body weight daily) through gavage; 4) Treatment Group: received GA at the dose of 100 mg/kg body weight daily through gavage. Treatments continued for two weeks.

**Results:**

Two weeks of GA treatment in diabetic mice reduced fasting blood glucose, improved plasma levels of hepatic enzymes, and increased liver tissue antioxidant capacity. Histopathological examination revealed that GA administration reduced diabetes-induced liver damage. Furthermore, GA treatment led to the down-regulation of Kelch-like ECH-associated protein 1 (Keap1) and up-regulation of nuclear factor E2-related factor 2 (Nrf2).

**Conclusion:**

The results of this study showed that GA exerts hepatoprotective effects in STZ-induced T2DM mice.

## Introduction

Type 2 diabetes mellitus (T2DM) is a prevalent metabolic disorder that leads to retinopathy, nephropathy, cardiomyopathy, and liver damage (1). Chronic liver disease is a primary cause of morbidity and mortality, and its prevalence is increasing globally (2). T2DM patients are at a high risk of liver failure, including nonalcoholic fatty liver disease, abnormal liver enzymes, hepatocellular carcinoma, cirrhosis, and acute liver failure (3, 4). The liver is the largest gland and one of the most vital organs, serving as a center for nutrient metabolism and waste metabolite excretion (5). Hyperglycemia, the hallmark of diabetes, is commonly associated with oxidative damage caused by reactive oxygen species (ROS) and the accumulation of lipid peroxidation by-products. The liver is one of the organs prone to oxidative stress caused by hyperglycemia, ultimately leading to liver damage. Therefore, oxidative stress contributes to the development of liver damage caused by diabetes (6). 

Nuclear factor E2-related factor 2 (Nrf2) is a transcription factor that regulates the expression of key components of the cell’s anti-oxidant system and thus plays a critical role in cellular redox homeostasis. Cells normally counteract the negative effects of oxidative stress by activating Nrf2. Kelch-like ECH-associated protein 1 (Keap1) is the most studied and probably the most important regulator of Nrf2. Keap1 is responsible for the constant ubiquitination and degradation of Nrf2 under homeostatic conditions. Keap1 is oxidized in response to oxidative stress, resulting in Keap1 inactivation and Nrf2 stabilization and translocation into the nucleus (7). One of the main target enzymes activated by Nrf2 is NAD(P)H: quinone oxidoreductase 1 (Nqo1), an intracellular cytosolic enzyme that catalyzes quinone reduction. It has been shown that Nqo1 induction is associated with decreased susceptibility to oxidative stress (8). 

Anti-oxidant compounds have a broad spectrum of biological activity. They can protect the body against free radicals and the destructive effects of ROS, in addition to reducing the progression of many chronic diseases by improving lipid peroxidation (9, 10). Gentisic acid (GA) is a metabolite of salicylates, and is also a phenolic compound abundant in natural sources such as gentian, citrus fruits, and olives (11); This compound is a highly effective anti-oxidant and free radical scavenger that shows anti-inflammatory, cardio-protective, hepato-protective, and anti-obesity properties (11, 12). In the previous studies, we showed that GA exerts antidiabetic and reno-protective effects (13, 14). Considering the role of the liver in regulating the homeostasis of carbohydrates and the fact that liver damage is one of the complications of diabetes, the possible protective effects of GA against liver damage caused by T2DM induced by nicotinamide (NA)-streptozotocin (STZ) have been investigated in this study.

## Materials and Methods

***Chemicals and Reagents***

Ketamine and xylazine were purchased from Alfasan Co (Netherlands). STZ, NA, metformin (MT), GA, and Bradford reagent were provided by Sigma-Aldrich (USA). Sodium hydroxide, hydrochloric acid, citric acid, and sodium citrate dihydrate were obtained from Merck (Germany).

***Animals***

This study was conducted on 24 adult male Naval Medical Research Institute (NMRI) mice in the weight range of 25 to 35 g. To acclimatize animals to the environment, one week before the study, all mice were transferred to the laboratory with 12-hour light and dark cycles and free access to food and water. All animal experiments were approved by Jundishapour University of Medical Sciences (Ahvaz, Iran) (IR.AJUMS.ABHC.REC.1401.070). 

***Study design and grouping***

**Figure 1:**
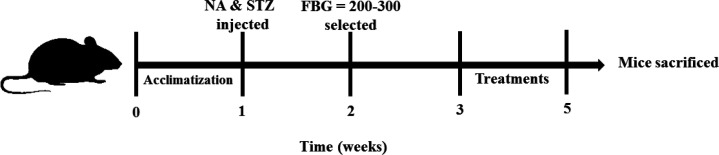
Schematic representation of experimental procedure

T2DM was established by intraperitoneal injection of a single dose of STZ (65 mg/kg), 15 min after the injection of NA (120 mg/kg). NA was dissolved in normal saline, and STZ in freshly prepared citrate buffer (pH 4.5) (15, 16). One week later, blood glucose levels (measured in the tail vein) were measured using a glucometer, and mice with fasting blood glucose (FBG) levels of 200-300 mg/dl were considered diabetic. The normal control mice were injected with vehicles instead of STZ and NA. One week after the induction of diabetes, treatments with GA and MT were started and continued for two weeks (17). Figure 1 schematically depicts the T2DM induction and treatment processes. The grouping was as follows:

1. Normal control group: received normal saline orally.

2. Diabetic control group (untreated): received normal saline orally.

3. Positive control group: received MT orally at a dose of 150 mg/kg body weight daily through gavage.

4. Treatment group: received GA orally at a dose of 100 mg/kg body weight daily through gavage.

***Sample collection***

One day after the last treatments, the animals were anesthetized by injection of ketamine (70 mg/kg body weight, IP) and xylazine (7 mg/kg body weight, IP). The liver tissue was taken immediately, cleaned with normal saline, and cut into pieces. One piece of the liver tissue was fixed in 10% formalin for histological examination, while the others were frozen in liquid nitrogen vapor and stored at -80 ^°^C for future use. Blood samples were collected from the left ventricles and centrifuged (3000 rpm, 10 min), then the obtained plasma was stored at -20 ^°^C for further tests. 

***Biochemical measurements***

In all plasma samples, alkaline phosphatase (Alk P), alanine transaminase (ALT), and aspartate aminotransferase (AST) were measured using commercial kits (Pars Azmoon, Co, Tehran, Iran).

***Evaluation of anti-oxidant enzymes and malondialdehyde (MDA)***

After homogenizing the liver tissues in a cold phosphate-buffered saline solution supplemented with a protease inhibitor cocktail (pH=7.4), the homogenates were centrifuged at 4000 rpm for 20 min at -4 ^°^C to collect the supernatants. MDA concentration, catalase (CAT) activity, superoxide dismutase (SOD) activity, and glutathione peroxidase (GPX) activity were all measured in the supernatants. All assessments were carried out according to the manufacturer’s instructions, using the colorimetry technique and related commercial kits (Zell Bio GmbH, Germany). The protein concentration of the samples was determined using the Bradford protein assay using identical supernatants. The protein concentrations in the samples were used to adjust all measurements.

***Histological analysis***

Mice liver tissues fixed in 10% formalin solution were dehydrated at a graded alcohol concentration and embedded in paraffin. 4-6 μm sections were prepared and stained with Hematoxylin and Eosin (H&E). The stained sections were used to evaluate the histopathological changes using a digital research microscope (BMZ-04-DZ, Behin Pajouhesh ENG. CO., Iran). 

***Quantitative real-time PCR analysis***

Briefly, after extracting tissue RNA according to the kit manufacturer’s instructions (Sina pure RNA extraction kit, Tehran, Iran), RNA purity was assessed using a nanodrop spectrophotometer. The isolated RNA was then converted to cDNA using a cDNA synthesis kit (cDNA Synthesis Kit, Anacell, Iran). Finally, the levels of Nrf2, Keap-1, and Nqo1 were analyzed by the StepOne real-time PCR system using the SYBR Green PCR master mix according to instructions of the manufacturer (High ROX; Amplicon, Denmark). To normalize the levels of Nrf2, Keap-1, and Nqo1, Glyceraldehyde-3-phosphate dehydrogenase (GAPDH) was used as an internal control. The ΔΔCt method was used to calculate the fold changes in mRNA levels in the different groups relative to the mean values in the control group. The specific primers used in this study are represented in Table 1.

***Statistical analysis***

SPSS software was used for all statistical analyses (version 24). All data sets were checked for assumptions required for building analysis of variance models prior to analysis. The normality of the distributions and equality of variances were checked by the Shapiro-Wilk test and Levene’s test, respectively. The one-way analysis of variance (ANOVA) with the least significant difference (LSD) *post hoc* test was used to analyze data sets that the variances between groups were equal. In every data set where variances between different groups were not equal, the corrected Welch’s one-way ANOVA and Games Howell *post hoc* test were used for data analysis. The non-parametric Kruskal-Wallis and the corresponding Dunn’s *post hoc* tests were used for the analysis of data that were not normally distributed. For parametric tests, the data were expressed as the mean±standard error of measurement (SEM), and for non-parametric tests, as the median±interquartile range (IQR). The level of significance was set at *P*<0.05. GraphPad Prism 6 was used for making graphs.

After one week of acclimatization, diabetes was induced by intraperitoneal injection of a single dose of streptozotocin (at the dose of 65 mg/kg), 15 min after the injection of nicotinamide (at the dose of 120 mg/kg). One week later, FBG levels were measured, and mice with FBG levels of 200-300 mg/dl were selected as diabetic. One week later, treatments with GA and MT started and lasted for two weeks. At the end of the fifth week mice were sacrificed

FBG: fasting blood glucose; GA: Gentisic acid; MT: metformin

**Figure 2:**
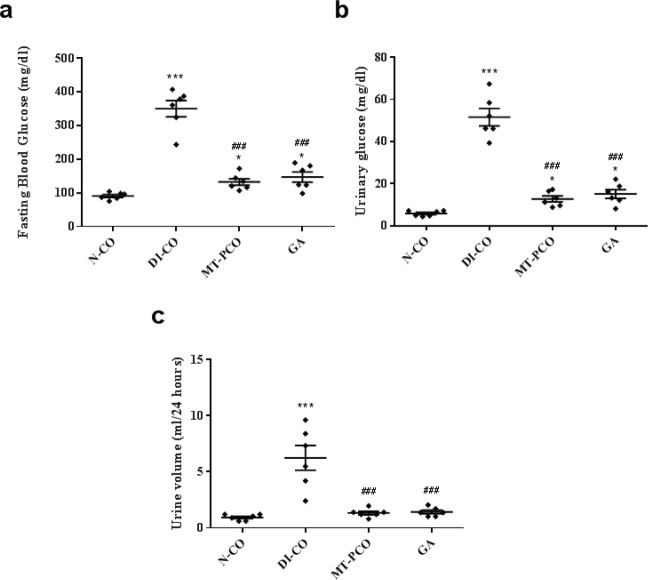
Effects of GA on a) fasting blood glucose (FBG), b) urinary glucose concentration, and c) 24 hr urine volume of mice in different experimental groups

Data are expressed as mean±SEM, n=6 in each group. *P<0.05, **P<0.01, and ***P<0.001 vs Normal Control Group. ^#^*P<*0.05, ^##^*P<*0.01, and ^###^*P<*0.001 vs Diabetic Control Group

N-CO: normal control group, DI-CO: diabetic control group, MT-PCO: metformin positive control group, and GA: Gentisic acid Group

**Table 1:** Forward and reverse sequences of primers used for Quantitative real-time PCR analysis of Keap-1, Nrf2 and Nqo1 gene expression

	Forward (5'-3')	Reverse (5'-3')
**GAPDH**	TGCTGGTGCTGAGTATGTCG	CGGAGATGATGACCCTTTG
**Keap-1**	TGCCCCTGTGGTCAAAGTG	GGTTCGGTTACCGTCCTGC
**Nrf2**	CTGAACTCCTGGACGGGACTA	CGGTGGGTCTCCGTAAATGG
**Nqo1**	ATTGTACTGGCCCATTCAGA	GGCCATTGTTTACTTTGAGC

**Figure 3:**
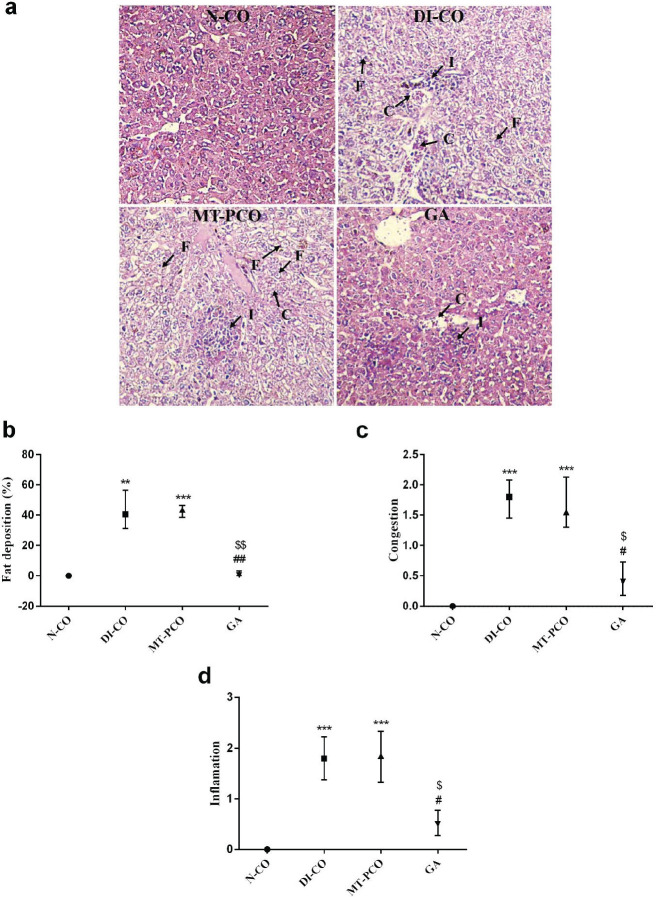
Effects of GA against liver tissue damages in T2DM mice

a) Images taken from H&E stained sections were shown with ×250 magnification (F: fat deposition, C: congestion and I: inflammation), b) Fat deposition, c) Congestion, and d) Inflammation. Data are represented in median±IQR (Interquartile range), n=6 in each group. * *P<*0.05, ** *P<*0.01, and ***P<0.001 vs Normal Control Group. ^#^*P<*0.05 and ^##^*P<*0.01 vs Diabetic Control Group. ^$^P<0.05 and ^$$^P<0.01 vs Metformin Positive Control Group

GA: Gentisic acid; T2DM: Type 2 diabetes mellitus

**Figure 4:**
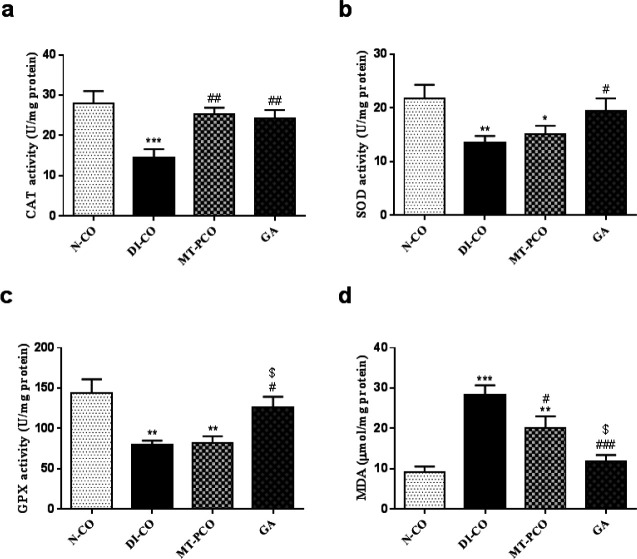
Effects of Gentisic acid (GA) on: a) Catalase activity, b) Superoxide dismutase activity, c) glutathione peroxidase activity, and d) MDA; malondialdehyde of mice liver tissue

Data are expressed as mean±SEM, n=6 in each group. *P<0.05, **P<0.01, and ***P<0.001 vs Normal Control Group. ^#^*P<*0.05, ^##^*P<*0.01, and ^###^*P<*0.001 vs Diabetic Control Group. ^$^*P<*0.05 vs Metformin Positive Control Group

**Table 2:** Effects of GA on the hepatic enzymes of mice in different experimental groups

Groups	Parameters		
	Alk P ^†^ (IU/L)	AST ^£^ (IU/L)	ALT ^¥^ (IU/L)
N-CO	126.84 ± 21.84	193.67 ± 14.8	29.5 ± 4.16
DI-CO	232.17 ± 31.93 ^**^	408 ± 22.18 ^***^	65 ± 11.47 ^**^
MT-PCO	287.17 ± 23.7 ^***^	211.84 ± 8.12 ^###^	31.84 ± 4.24 ^##^
GA	170.66 ± 10.67 ^$$^	197.5 ± 22.7 ^###^	28.83 ± 5.91 ^##^

Data are expressed as mean±SEM (n=6). † alkaline phosphatase. £ aspartate aminotransferase. ¥ alanine transaminase

*P<0.05, **P<0.01, and ***P<0.001 vs Normal Control Group. ^#^*P<*0.05, ^##^*P<*0.01, and ^###^*P<*0.001 vs Diabetic Control Group. ^$$^*P<*0.01 vs Metformin Positive Control Group

N-CO: Normal Control Group, DI-CO: Diabetic Control Group, MT-PCO: Metformin Positive Control Group and GA: Gentisic acid Group

**Figure 5:**
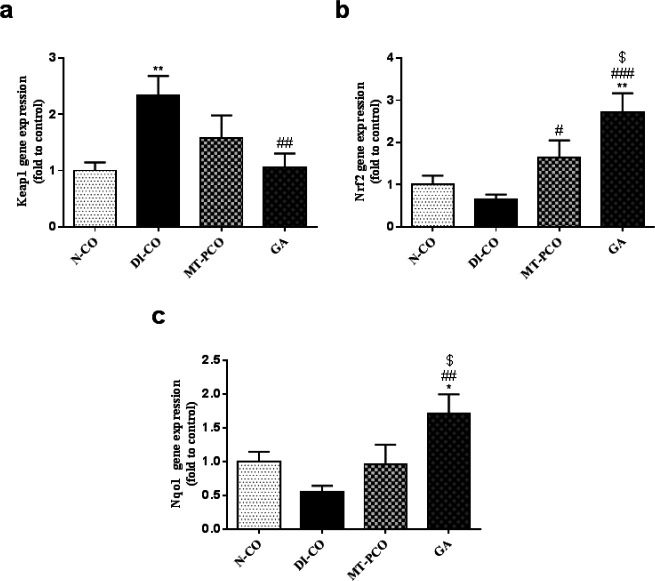
Effects of Gentisic acid (GA) on a) Keap1, b) Nrf2, and c) Nqo1 relative expressions of mice liver tissue

Data are expressed as mean±SEM, n=6 in each group. *P<0.05, **P<0.01, and ***P<0.001 vs Normal Control Group. ^#^*P<*0.05, ^##^*P<*0.01, and ^###^*P<*0.001 vs Diabetic Control Group. ^$^*P<*0.05 vs Metformin Positive Control Group

## Results

***Effect of GA on FBG, urinary glucose levels, and urinary output***

After one week of T2DM induction, the FBG significantly increased compared with normal mice (Figure 2a) accompanied by a significant increase in urine glucose levels (Figure 2b) and polyurea (Figure 2c). These results showed that T2DM was successfully induced. Administration of GA and MT to the diabetic mice significantly decreased FBG, urinary glucose, and urine volume compared with untreated diabetic mice (Figure 2). 

***Effect of GA on histopathological changes in the liver tissues***

The histopathological changes of liver tissues induced by T2DM were evaluated by liver tissue sections stained by H&E, as shown in Figure 3a. T2DM is associated with NAFLD, which leads to increased fat deposition in the hepatocytes (18). At the end of the experiment, histopathological analysis showed that diabetes induction led to a significant increase in fat deposition in the liver tissue. Interestingly, treatment of diabetic mice with MT failed to ameliorate increased fat deposition in hepatocytes. On the contrary, treatment with GA restored the percentage of fat deposition in the diabetic mice to the level of normal control mice (Figure 3a and b). In addition to increased fat deposition, T2DM induces hepatic congestion and inflammatory cell infiltration (19). Two-week GA administration to the diabetic mice led to a significant decrease in hepatic congestion and inflammation compared to DI-CO and MT-PCO groups (Figure 3a, c, and d).

***Effect of GA on plasma Alk-P, AST, and ALT levels***

As stated earlier, T2DM contributes to liver disease. Elevated levels of Alk-P, AST, and ALT in the blood are signs of liver damage. So, the plasma levels of these enzymes in the different experimental animals were evaluated in this study. As illustrated in Table 2, the levels of Alk-P, AST, and ALT in the plasma of diabetic mice were significantly increased compared with intact mice. MT and GA treatment of diabetic mice prevented AST and ALT elevation in plasma; however, MT significantly increased Alk-P levels even when compared to the diabetic control group. However, GA successfully restored plasma Alk-P to normal mice levels. 

***Effect of GA on hepatic anti-oxidant enzymes and MDA levels***

GPX, SOD, and CAT activities were significantly decreased in the liver tissue of diabetic mice as compared to the N-CO group. Diabetic mice treated with GA showed a significant improvement in the liver’s CAT, SOD, and GPX activities. MT significantly increased CAT activity compared with the diabetic control group. No significant differences were observed in SOD and GPX activity between the DI-CO group and MT-PCO mice (Figure 4a, b, and c).

The liver tissue of diabetic mice showed a significant elevation in MDA content as compared to the N-CO group. Treatment with GA and MT significantly ameliorated the elevated MDA levels in the liver of diabetic mice (Figure 4d).

***Effect of GA on liver Keap1, Nrf2, and Nqo1 relative mRNA expression***

The Keap1-Nrf2 system is involved in many biological processes. Nrf2 is the most important regulator of genes associated with cells’ responses against oxidative stress. Keap1 is the cytosolic repressor and the negative regulator of the Nrf2 protein (20). Nqo1 is one of the main target genes of Nrf2, and its increased expression is highly correlated with increased expression and the activity of the Nrf2 protein (21). So, we evaluated the relative mRNA expression of Keap1, Nrf2, and Nqo1 in this study. As shown in Figure 5a, the Keap1 mRNA expression significantly increased in the diabetic mice, and consequently, the Nrf2 mRNA expression in this group decreased; however, this decrease was not statistically significant (Figure 5b). GA administration to the diabetic mice caused a significant decrease in Keap1 mRNA expression compared with the untreated diabetic mice, and as a result, Nrf2 expression significantly increased in this group (Figure 5a and b). Also, Nqo1 mRNA expression was significantly up-regulated in GA-treated T2DM mice compared to the other groups, and this increase can be caused by increased Nrf2 expression and activity in the GA-treated diabetic mice (Figure 5c).

## Discussion

Diabetes is a chronic metabolic disorder with many complications that lead to reduced quality of life and increased mortality in diabetic patients. One of these complications is liver injury, which is very important due to the organ’s crucial role in lipid and carbohydrate homeostasis (22). In this study, a non-obese T2DM model was established by intraperitoneal injection of STZ after NA injection. STZ exerts cytotoxic effects on pancreatic beta cells, which ultimately leads to decreased insulin secretion. However, there is evidence that STZ also has direct toxic effects on other cells, including the hepatic cells (23, 24). On the other side, NA is a cytoprotective compound, so we administered it before STZ injection to protect the liver tissue from the direct cytotoxic effects of STZ. One week after STZ injection, FBG, glucose excretion in the urine, and urine volume significantly increased. Diabetic mice treated with GA or MT had lower FBG levels, urinary glucose levels, and urine volume. 

The plasma Alk-P, AST, and ALT levels were determined in this study to evaluate liver function. AST and ALT are the hepatocytes’ cytosolic and mitochondrial enzymes, and the elevated plasma levels of these enzymes are the primary indicators of cellular damage in the liver tissue (25). A positive correlation between plasma levels of AST and ALT and diabetes mellitus has been reported in previous studies (26, 27). An elevated plasma Alk-P level is also an indicator of abnormal liver function (28). Jing *et al*. (2020), in a rat model of T2DM, reported that diabetes induction led to a significant increase in AST and ALT, and treatment of diabetic rats with pterostilbene, which is a natural polyphenolic compound, significantly decreased AST and ALT levels (29). Another study, reported that in a D-galactoseamine-induced hepatic injury in rats, treatment with carvacrol, a polyphenolic compound, restored Alk-P, AST, and ALT to their normal levels (30). In line with these findings, we observed increased plasma levels of AST and ALT in STZ-induced T2DM mice compared with the normal control group. GA and MT prevented diabetes-induced AST and ALT elevations in the plasma. Here we showed that plasma Alk-P level significantly increased upon diabetes induction; no improvement was observed about this enzyme in diabetic mice treated with GA or MT; however, Alk-P level was higher in MT-treated animals, and GA compared with the MT group significantly decreased Alk-P levels. 

Histopathological analysis showed abnormal hepatic cell architecture, congestion, inflammation, and increased fat deposition in the liver tissue of diabetic mice. These results are in agreement with the previous studies (31, 32). Dyslipidemia is well-documented in diabetic patients (33). In diabetics, due to lack of insulin or impaired insulin actions because of insulin resistance, the elevated blood glucose fails to catabolize, and as a result, glucose converts to fatty acids in the liver. Consequently, fat deposition in the liver increases, which per se exacerbates insulin resistance (34). The increased fatty acid synthesis and increased fat deposition in hepatic tissue induce oxidative stress and inflammation that ultimately lead to liver injury (35). Hewage *et al*. (2021) reported that lingonberry, which possesses anti-oxidant and anti-inflammatory properties, attenuates liver injury induced by a high-fat diet in mice by decreasing hepatic fat deposition, oxidative stress, and inflammation (36). A study showed that oral administration of GA in high-fat diet-fed mice decreased body weight, improved insulin sensitivity, and reduced systemic lipid accumulation (12). Consistent with these observations, in the present study we showed that GA treatment decreased hepatic fat deposition and inflammation and restored the normal morphology of the liver in comparison with the diabetic control mice. Interestingly, MT treatment has not been effective in improving liver histology. Since both GA and MT lowered FBG, it can be concluded that the protective effects of GA against diabetes-induced liver injury are independent of its blood glucose-lowering effect. 

It is well established that oxidative stress contributes to the pathogenesis and progression of liver disease (37-40). Hyperglycemia is associated with increased ROS production and decreased tissue anti-oxidant capacity (41). The liver, as an important insulin-sensitive tissue, is one of the primary organs vulnerable to the oxidative stress induced by hyperglycemia, which ultimately results in liver tissue injury (38). Several studies have demonstrated the beneficial effects of compounds with anti-oxidant effects for the prevention of diabetes-associated complications (42-44). The Keap1-Nrf2 system is essential for the cell’s ability to defend against oxidative stress. Nrf2 is a transcription factor that is involved in the induction of anti-oxidant and phase II detoxifying enzymes (20). So, Nrf2 activation is a strategy to potentiate the tissue’s anti-oxidant capacity for protection against oxidative stress-induced damage. Mahmoud *et al*. (2020) showed that ferulic acid, as a natural anti-oxidant, ameliorated methotrexate-induced liver injury in rats by up-regulating Nrf2 protein expression (45). Researchers, in a mouse model of nonalcoholic fatty liver disease induced by a high-fat and high-fructose diet, showed that treatment with curcumin, which is a polyphenolic compound, improved liver injury by modulating the Nrf2 pathway (46). Also, GA is a potential Nrf2 activator, which may account for its anti-oxidant effects (47). Consistent with these reports, in this study, we showed that the Keap1 mRNA level significantly decreased upon treatment with GA, which led to increased Nrf2 expression. Increased Nqo1 mRNA levels in GA-treated mice confirmed increased Nrf2 expression and activity. Anti-oxidant enzymes such as SOD, CAT, and GPX are the downstream targets of Nrf2, which play a pivotal role in detoxifying free radicals. Previous studies have found that tissue anti-oxidant capacity is reduced in STZ-induced diabetes animal models (48-50). In line with these findings, the measurement of anti-oxidant enzymes revealed that diabetes induction by STZ leads to a significant decrease in the activity of CAT, SOD, and GPX enzymes in the liver tissue. Also, MDA levels have been evaluated in this study as an indicator of oxidative stress, and an increase in this marker was observed in diabetic mice. Further treatment of the diabetic mice with GA increased the activity of anti-oxidant enzymes as well as reducing lipid peroxidation, thereby attenuating oxidative stress in the liver tissue. As a result, we observed a shift in the oxidant/anti-oxidant balance in favor of anti-oxidant enzymes, which could lead to the amelioration of liver tissue damage. 

## Conclusion

We observed for the first time the hepatoprotective effects of GA in STZ-induced T2D mice. GA improved the anti-oxidant status capacity of the liver tissue, ameliorated liver damage biomarkers, and improved the histopathological alterations induced by diabetes. Nrf2 mRNA was up-regulated upon treatment of the diabetic mice with GA, and this is the most likely relevant mechanism of its protective effect against liver injury.

## Authors’ Contributions

A A, R N R and E H conceived and designed the project. R N R, E H, N N, M J S, and A A performed experiments, data processing, and collection. A A and R N R analyzed and interpreted the results. A A, R N R, and E H prepared the draft manuscript and visualization; A A revised and edited the article. A A provided supervision and funding acquisition. R N R, E H, N N, M J S, and A A approved the final version to be published. 

## Conflicts of Interest

The authors have no relevant financial or non-financial interests to disclose.
